# Efficient focusing of 8 keV X-rays with multilayer Fresnel zone plates fabricated by atomic layer deposition and focused ion beam milling

**DOI:** 10.1107/S0909049513006602

**Published:** 2013-04-09

**Authors:** Marcel Mayer, Kahraman Keskinbora, Corinne Grévent, Adriana Szeghalmi, Mato Knez, Markus Weigand, Anatoly Snigirev, Irina Snigireva, Gisela Schütz

**Affiliations:** a Modern Magnetic Systems, Max Planck Institute for Intelligent Systems, Heisenbergstrasse 3, D-70569 Stuttgart, Germany; b Institut Für Angewandte Physik, Friedrich-Schiller-Universität Jena, Albert-Einstein-Strasse 15, D-07745 Jena, Germany; c CIC nanoGUNE Consolider, Tolosa Hiribidea 76, E-20018 Donostia-San Sebastian, Spain; d Ikerbasque, Basque Foundation for Science, Alameda Urquijo 36-5, E-48011 Bilbao, Spain; e European Synchrotron Radiation Facility, 6 rue Jules Horowitz, BP 220, F-38043 Grenoble, France

**Keywords:** sputtered-sliced, multilayer, Fresnel zone plate, hard X-ray, ALD, FIB

## Abstract

Fresnel zone plates (FZPs) recently showed significant improvement by focusing soft X-rays down to ∼10 nm. In contrast to soft X-rays, generally a very high aspect ratio FZP is needed for efficient focusing of hard X-rays. Therefore, FZPs had limited success in the hard X-ray range owing to difficulties of manufacturing high-aspect-ratio zone plates using conventional techniques. Here, employing a method of fabrication based on atomic layer deposition (ALD) and focused ion beam (FIB) milling, FZPs with very high aspect ratios were prepared. Such multilayer FZPs with outermost zone widths of 10 and 35 nm and aspect ratios of up to 243 were tested for their focusing properties at 8 keV and shown to focus hard X-rays efficiently. This success was enabled by the outstanding layer quality thanks to ALD. *Via* the use of FIB for slicing the multilayer structures, desired aspect ratios could be obtained by precisely controlling the thickness. Experimental diffraction efficiencies of multilayer FZPs fabricated *via* this combination reached up to 15.58% at 8 keV. In addition, scanning transmission X-ray microscopy experiments at 1.5 keV were carried out using one of the multilayer FZPs and resolved a 60 nm feature size. Finally, the prospective of different material combinations with various outermost zone widths at 8 and 17 keV is discussed in the light of the coupled wave theory and the thin-grating approximation. Al_2_O_3_/Ir is outlined as a promising future material candidate for extremely high resolution with a theoretical efficiency of more than 20% for as small an outermost zone width as 10 nm at 17 keV.

## Abstract 2

The fabrication and performance of multilayer Al_2_O_3_/Ta_2_O_5_ Fresnel zone plates in the hard X-ray range and a discussion of possible future developments considering available materials are reported.

## 1. Introduction

X-ray microscopy is an invaluable tool for many scientific and technological fields such as, for instance, material (Zschech *et al.*, 2011 ▶), environmental (Brown & Sturchio, 2002 ▶) and life sciences (Lewis, 1997 ▶; Fouras *et al.*, 2009 ▶). The utilization of focused X-rays has spread continuously over recent decades and includes applications such as imaging, chemical analysis (Sakdinawat & Attwood, 2010 ▶) and time-resolved investigation by exploiting pulses of synchrotron radiation (Kammerer *et al.*, 2011 ▶; Van Waeyenberge *et al.*, 2006 ▶; Saes *et al.*, 2003 ▶). In contrast to electron microscopy, X-ray microscopy provides high penetration depth and enables three-dimensional imaging of complete cells (Vogt *et al.*, 2000 ▶), large alive biological samples (Olendrowitz *et al.*, 2012 ▶) and *in situ* investigation under extreme conditions (Fife *et al.*, 2012 ▶). As the theoretical resolution is determined by the wavelength, hard X-ray microscopy is a very promising technique for high-resolution imaging with chemical sensitivity (Schroer *et al.*, 2004 ▶). Unfortunately, the focusing optics for hard X-rays are limited in terms of resolution and/or diffraction efficiency (DE). Conventionally, Kirkpatrick–Baez mirrors (KBMs) are used for focusing hard X-rays despite difficulties in manufacturing and high costs, and routinely achieve two-dimensional resolutions in the range of a few hundreds of nano­meters (Ruhlandt *et al.*, 2012 ▶; Riekel *et al.*, 2009 ▶). Higher resolutions were also reported with advanced wavefront-correction techniques (Mimura *et al.*, 2010 ▶, 2011 ▶; Yamauchi *et al.*, 2011 ▶). Yet another example of advanced KBMs is based on a set of four mirrors, two elliptical and two hyperbolic, which achieved approximately 50 nm resolution (Matsuyama *et al.*, 2012 ▶). Other approaches for two-dimensional focusing of X-rays include using, for instance, crossed nanofocusing lenses [below 50 nm foreseen (Schroer *et al.*, 2010 ▶), 80 nm reported (Schropp *et al.*, 2012 ▶)], crossed multilayer Laue lenses (MLLs) (Yan *et al.*, 2011 ▶) or compound refractive lenses (Lengeler *et al.*, 2005 ▶) with potential for high energies up to 100 keV (Vaughan *et al.*, 2011 ▶). In one dimension X-rays could be focused down to 50 nm (Matsuyama *et al.*, 2010 ▶) with one-dimensional Wolter optics and to 16 nm (Kang *et al.*, 2008 ▶) and 7.5 nm (Ruhlandt *et al.*, 2012 ▶) using single MLLs. The lack of high-performance optics for hard X-rays led to lensless microscopy (Falcone *et al.*, 2011 ▶) which requires high coherence. Lensless techniques can benefit from a high flux *via* focusing optics, enabling an increased coherent flux density, improving the resolution (Schropp *et al.*, 2012 ▶).

Fresnel zone plates (FZPs) (Falcone *et al.*, 2011 ▶) provided the highest two-dimensional resolutions for soft X-rays (Chao *et al.*, 2005 ▶, 2009 ▶; Vila-Comamala *et al.*, 2009 ▶). Their resolution at the first-order focus is determined by the width of the outermost zone, Δ*r*_*n*_, *via* the relation *r*_Rayleigh_ = 1.22Δ*r*_*n*_, where *r*_Rayleigh_ is the Rayleigh resolution (Attwood, 2000 ▶). It follows from this relation that decreasing Δ*r*_*n*_ increases the resolution. Electron beam lithography (EBL), the conventional method for FZP fabrication, enabled fabrication of ever smaller values of Δ*r*_*n*_. Although FZPs fabricated *via* EBL-based methods are presently employed for focusing hard X-rays, the limitation in achievable aspect ratios (*A*_*r*_ = *t*/Δ*r*_*n*_, where *t* is the thickness) of current FZPs limits their diffraction efficiencies (Chen *et al.*, 2008 ▶; Chu *et al.*, 2008 ▶; Vila-Comamala *et al.*, 2012 ▶).

In this work we report the preparation and testing of high-aspect-ratio FZPs with high efficiency in the hard X-ray range. The new fabrication method employed delivers multilayer FZPs (ML-FZPs) and implicates the use of atomic layer deposition (ALD) and focused ion beam (FIB) milling (Mayer *et al.*, 2011 ▶). ML-FZPs (also called ‘sputtered-sliced’ FZPs) were introduced in the 1980s as an alternative approach to fabricate FZPs with high *A*_*r*_ (Rudolph & Schmahl, 1980 ▶; Rudolph *et al.*, 1981 ▶). Unfortunately, three decades after its introduction, and despite the fact that the approach has been pursued by various groups (Bionta & Skulina, 1993 ▶; Golant *et al.*, 2007 ▶; Koyama *et al.*, 2011 ▶; Liese *et al.*, 2011 ▶; Tamura, 2011 ▶), ML-FZPs failed to achieve the anticipated high resolution and DE for hard X-rays, as fabrication techniques failed to meet the required high precision. Recently, our group fabricated a high-quality ML-FZP by combining ALD and FIB and demonstrated the resolution of sub-39 nm structures at 1.2 keV (Mayer *et al.*, 2011 ▶), the highest resolution achieved by a ML-FZP to the best of our knowledge. The method consists of coating a wire with alternating layers of two materials of proper refractive indices. Subsequent slicing/polishing delivers the ML-FZP of desired thickness *t*. As there is no limitation to the thickness *t* of a ML-FZP, the method is especially suited for fabricating high-*A*_*r*_ FZPs. While ALD gives atomic control over Δ*r*_*n*_, *i.e.* resolution, *via* highly conformal layers around the substrate with very high quality interfaces, FIB slicing of the multilayer ensures local control, a precise cut and preserves the integrity of the layers. Focusing properties of the ML-FZPs with Δ*r*_*n*_ = 35 and 10 nm were tested at 8 keV, from which experimental DEs were estimated. Estimates were compared with the results of the thin-grating (TG) approximation (Kirz, 1974 ▶; Yun *et al.*, 1992 ▶) within the framework of scalar diffraction theory and considering the volume effects described by coupled wave theory (CWT) (Schneider *et al.*, 2008 ▶) using published data for complex refractive indices [CXRO, LBNL, USA (Henke *et al.*, 1993 ▶)]. Scanning transmission X-ray microscopy (STXM) with one of the FZPs with Δ*r*_*n*_ = 35 nm was also performed at 1.5 keV. Following theoretical calculations and fabrication prospects, issues concerning the choice of material for ML-FZPs at 8 and 17 keV for future developments are discussed.

## 2. Experimental method

**Figure 1:**
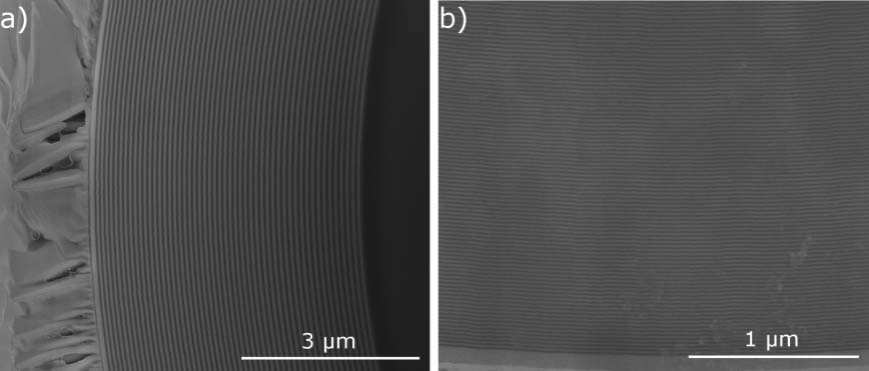
Scanning electron micrographs of ML-FZPs with Δ*r*_*n*_ = 35 nm (ZP35) (*a*) and 10 nm (ZP10) (*b*). Note the high quality of the zones.

Commercial glass optical fibers (A2 by SCHOTT AG, Germany; *D* = 30 µm) were coated with alternating layers of Al_2_O_3_ and Ta_2_O_5_ *via* ALD following the zone-plate law. The total layer thickness was 4 µm, composed of 103 to 360 zones, resulting in ML-FZPs with 38 µm diameter and an active focusing region that is 37.7% of the total area. ML-FZPs with Δ*r*_*n*_ = 35 and 10 nm were prepared; their parameters and names are given in Table 1 ▶. The resulting multilayer structures were sliced and polished using FIB (Nova NanoLab, FEI, The Netherlands) to deliver the ML-FZPs which were mounted on molybdenum TEM grids for easy handling. According to CWT calculations the required thickness for the best efficiency, *t*_Optimum_, of ZP10 is 2.45 µm whereas it is 8.85 µm for a ML-FZP with Δ*r*_*n*_ = 35 nm. The realised thickness, *t*_Real_, of the ZP10, ZP35t and ZP35opt ML-FZPs were ∼1.9, ∼5.9 and ∼8.5 µm, respectively. *A*_*r*_ values of ML-FZPs here are extremely high (169 for ZP35t, 243 for ZP35opt and 190 for ZP10) when compared with zone plates used for soft or hard X-ray microscopy prepared by e-beam lithography, the maximum of which is 20–25 (Vila-Comamala *et al.*, 2010 ▶, 2012 ▶). Figs. 1(*a*) and 1(*b*) ▶ show scanning electron micrographs of ZP35t and ZP10, respectively. The high quality of the layers and layer interfaces over large distances can be seen. No beamstops were deposited on the ML-FZPs or otherwise employed in the experiments. Further details of the fabrication can be found elsewhere (Mayer *et al.*, 2011 ▶).

**Figure 2:**
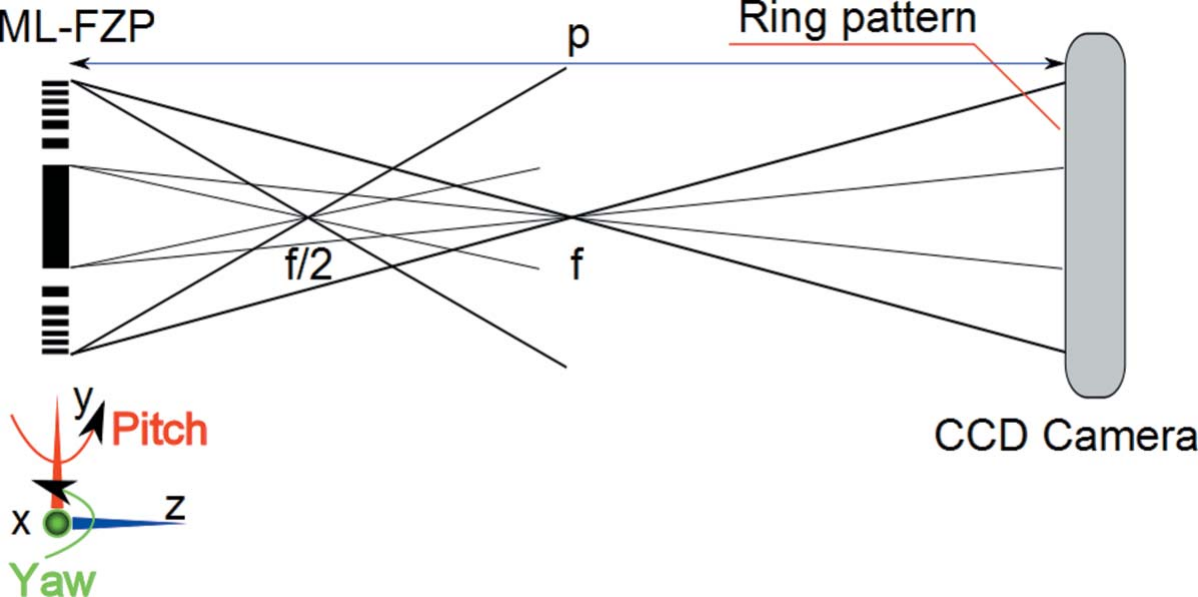
Schematic representation of the experimental set-up that was used at beamline ID06 of the ESRF for focusing and DE measurements (not to scale).

Focusing tests at 8 keV were carried out at beamline ID06 of ESRF (Grenoble, France). In these tests the ML-FZPs were mounted on precision translation and rotation stages (HUBER Diffraktionstechnik GmbH, Germany) downstream of the X-rays. A CCD camera with 1376 × 1040 pixels and 6.45 µm pixel size (Sensicam QE, PCO AG, Germany) equipped with a scintillator, which converts X-rays into visible light, and an objective lens (Olympus, UPLAPO) with 10× magnification were utilized as the detector. Hence, the resolution is 1.3 µm (2 pixels) and the field of view is 887 µm × 670 µm. First, the camera and ML-FZP were centred on the optical axis. Then, the sample stage and camera were moved towards each other until a section of or a complete bright ring appears in the projection image of the zone plate on the CCD camera. The bright ring consists of light which is diffracted from the active zones of the ML-FZP. At the same time as the bright ring appears, the area on the zone plate from which the light is diffracted away appears dark in the image. If only a section of a ring appears, the inclination between zone plate and beam has to be corrected *via* rotation about the pitch and yaw axes (Fig. 2 ▶) until the ML-FZP surface is perfectly perpendicular to the beam. When aligned to the optical axis, the active zones of the ML-FZP form a hollow cone of diffracted light which narrows towards the focal length (projection distance *p* < *f*; see Fig. 2 ▶), reaches a spot with minimum diameter at the focal length (*p* = *f*) and then diverges again (*p* > *f*). In the case of ZP35t and ZP35opt (Δ*r*_*n*_ = 35 nm), the first- and second-order focus could be reached (*p* = *f* and *p* = *f*/2, respectively) whereas in the case of ZP10 (Δ*r*_*n*_ = 10 nm) it was not possible to approach the camera close enough to be able to reach the first-order focal spot as the camera body prevented it coming closer to the ML-FZP (*p* > *f*). The evaluation of DE from the focusing experiments was performed according to the following procedure: the amount of incident light on the FZP (*I*_i_) was determined by multiplying the count density from an un­obstructed area in the vicinity of the FZP by the active area of the FZP. Then, a background-subtraction algorithm was employed (Sternberg, 1983 ▶; Bleiner *et al.*, 2011 ▶) to subtract the effect of radiation transmitted through the glass core prior to determination of the total light intensity in the diffraction pattern (*I*_f_). The ratio *I*_f_/*I*_i_ gives the estimated DE. It should be noted that the method employed here is not exact but should be taken only as an estimate owing to difficulties in separating the background intensity from that of the first-order focus. In the case of ZP35opt, which was tested on three occasions in two synchrotron experiments, the DE was estimated by taking all experiments into account.

In order to further evaluate the performance of the ML-FZPs as focusing optics for X-ray microscopy, ZP35t was also tested in a dedicated X-ray scanning transmission microscope at 1.5 keV (MAXYMUS, BESSYII, Berlin, Germany) by imaging a standardized Siemens star as a test object (X30-2-2, Xradia, USA). The MAXYMUS is a highly stable microscope which allows for very high resolution imaging (Follath *et al.*, 2010 ▶).

## 3. Experimental results and discussion

**Figure 3:**
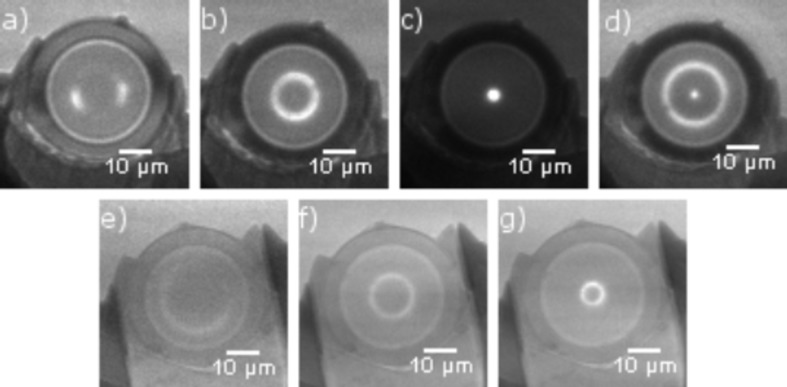
Diffraction experiments at 8 keV. Top row: far-field image of ZP35t on camera: (*a*) misaligned and defocused, (*b*) aligned and defocused, *p* > *f*, (*c*) camera in first-order focus, *p* = *f* (contrast enhanced for visibility), and (*d*) camera in second-order focus, *p* = *f*/2. Bottom row: far-field projection of ZP10 on camera: (*e*) misaligned and far from focus, (*f*) aligned and overfocused, *p* >> *f*, and (*g*) camera as close to the focus as the set-up permits, *p* > *f*.

After alignment of the ML-FZPs perpendicular to the X-ray beam by tuning pitch and yaw axes the initial far-field projection of the ML-FZPs on the CCD [Figs. 3(*a*) and 3(*e*) ▶] transformed into circularly symmetric diffraction rings [Figs. 3(*b*) and 3(*f*) ▶, *p* > *f*]. The symmetry of the diffraction pattern is a result of the high overall quality of the ML-FZPs and shows that all portions of the optical device function properly. When the distance *p* was decreased, the radii of the diffraction rings became proportionally smaller until a focal spot was observed on the camera at the focal distance. In the cases of ZP35t and ZP35opt, it was possible to find the focal plane (within the precision of the set-up) as shown in Fig. 3(*c*) ▶, *p* = *f*. The diffraction pattern at position *p* = *f* (first-order focal spot) is spread over several pixels owing to pixel cross-talk and the grained structure of the scintillator. As *p* was reduced further, the diameter of the first-order focal ring increased again and, as it approached *f*/2, it was possible to observe the second-order focus and underfocused first-order diffraction ring (*p* = *f*/2) on the same CCD image (Fig. 3*d* ▶). The activation of the second diffraction order, which should not exist for a perfect rectangular standard FZP, is a result of deviations from the ideal FZP. Controlling this effect can be used for higher-order imaging for increased resolution (Baciocchi *et al.*, 1994 ▶; Yi *et al.*, 2011 ▶). Here, this activation could be a result of the non-ideal zone profile, a line-to-space ratio that is different from 1:1 and a non-ideal zone placement or a combination thereof. In the same way after alignment the diffraction pattern of ZP10 appears as a ring on the screen of the camera [Figs. 3(*f*) ▶ and 3(*g*)] and is again a sign of the good quality of and symmetry of the zone plate. In these experiments all three ML-FZPs were shown to behave like a lens and focus 8 keV hard X-rays as expected.

**Figure 4:**
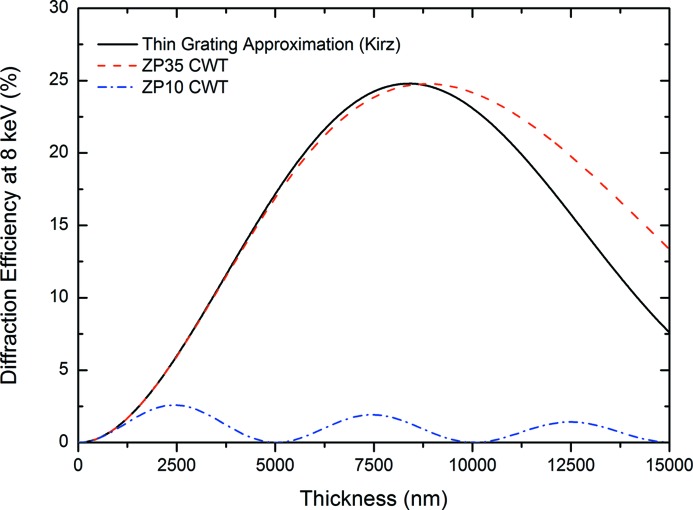
Theoretical efficiencies of ML-FZPs made out of Al_2_O_3_ and Ta_2_O_5_ with different Δ*r*_*n*_ as a function of thickness. CWT parameters were: zones parallel to optical axis, magnification of 10000×, line-to-space ratio 1:1.

The experimental DE of the ZP35t was estimated to be 15.58% *versus* 20.14% as predicted by the CWT which means that more than 77% of the theoretical DE was reached at 8 keV photon energy. ZP35opt was found to be 11.93% efficient with a standard deviation of 2.82% in three different measurements exhibiting reasonable repeatability. This corresponds to 48% of the theoretical DE which is lower than the ZP35t sample and will be discussed below. The measured DE of ZP10 was 1.87% which corresponds to about 81% of the theoretically predicted DE of 2.31%, according to CWT (see Fig. 4 ▶). High performances of the ML-FZPs were enabled by the outstanding quality of the layers as a result of the ALD process. From Fig. 4 ▶, it can be seen that the theoretical DEs of a ML-FZP with Δ*r*_*n*_ = 35 nm according to the CWT and TG approximation are very close even at very large aspect ratios. CWT and TG approximations give especially close estimations of efficiencies when the difference in photon wavelength and Δ*r*_*n*_ is large and the thickness of the structure is lower than the optimum thickness (see Fig. 4 ▶).

The reason for the difference in the abilities of ZP10, ZP35t and ZP15opt to approach their respective theoretical DE may be speculated to be the different thicknesses of the ML-FZPs. The thicker ML-FZPs ZP35t and ZP35opt are more sensitive to perfect alignment of the FZP and the structural irregularities that may be present in the propagation direction of the X-rays. The accumulated impact of these errors may be reflected by a reduced performance as the thickness increases. Although in-depth investigations should be carried out to find the exact reason for these differences, the effect of thickness should be taken into account when selecting a proper material pair for the design of a ML-FZP as *t*_Optimum_ for any candidate material should be as thin as possible in order to reduce the impact of imperfection on performance.

**Figure 5:**
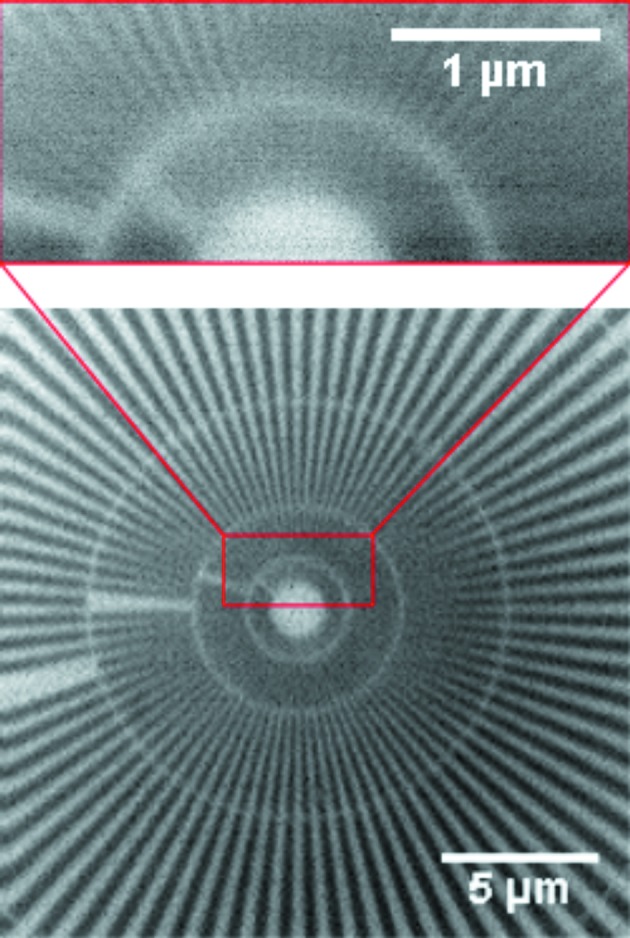
Imaging at 1.5 keV. Bottom: overview STXM image of a Siemens star showing inner structures (100 nm pixel size, 10 ms dwell time). Top: high-resolution image of a portion of the two innermost rings with the zoomed area marked in the overview (20 nm pixel size, 15 ms dwell time). 60 nm structures (120 nm period) of the second ring were resolved.

Taking advantage of its relatively low thickness, ZP35t was also tested at a lower photon energy (1.5 keV) in order to gain an insight into the focusing properties of the ML-FZP at the scanning X-ray microscope MAXYMUS. The STXM images of a Siemens star test object were recorded using ZP35t as the objective lens (Fig. 5 ▶). The spikes in the second ring from the center have smallest local periods of 120 nm which were clearly resolved. Theoretically, the resolution of this ML-FZP should have been ∼43 nm full period according to the Rayleigh criterion. Interestingly, in spite of attempts to resolve the innermost ring with 60 nm smallest local period, the achieved resolution was about 120 nm period, *i.e.* 60 nm feature size. This was presumably due to the low signal-to-noise ratio which was in turn caused by the very low DE of ZP35t at 1.5 keV (0.56%, theoretically) and the absence of a beamstop. The core of the ML-FZP which consists of silica glass of 5.9 µm thickness has a transmission of more than 20% at 1.5 keV which causes strong background radiation. Combined effects of a low DE and the absence of a beamstop resulted in the pronounced noise in the image shown in Fig. 5 ▶. It is believed that this increased noise caused the smallest structures to remain unresolved. When the STXM images in Fig. 5 ▶ are closely inspected, an apparent astigmatism is observed that is a variation in resolution in different directions. This could be due to several reasons, one of which is the misalignment of ZP35t from the optical axis as there were no possibilities to correct any misalignment at the MAXYMUS set-up. Owing to the very high aspect ratio of the ML-FZP, any misalignment from the optical axis may also cause a reduction in the numerical aperture due to a reduction of the effective active zone area leading to deterioration of the resolution in certain directions. A tilt stage which can be fit into the MAXYMUS to correct for possible misalignments is currently under development and will be available in the next months. Astigmatism may also originate from the variation of deposition thickness along and around the fiber which is very challenging to characterize in three-dimensions and must be investigated in more depth. The circularity of the core fiber as a possible source of astigmatism is excluded since it was already shown in a previous study that the fiber is cylindrical enough to be used as a substrate for a ML-FZP (Mayer *et al.*, 2011 ▶).

A 30 µm-wide beamstop as a central obstruction corresponds to 62% of the surface area of the lens, which leads to a significant apodization effect and resultant transfer of energy to the side-lobes of the Airy pattern (Simpson & Michette, 1984 ▶) with an accompanied shift of first zero-crossing radius of the Airy pattern, which is the definition of Rayleigh resolution (Attwood, 2000 ▶), to approximately 32.7 nm instead of 42.7 nm. Any negative or positive impact of this effect on the presented ML-FZPs remains to be confirmed.

Up to this point it has been shown that the ML-FZPs investigated in this work function properly at 8 keV with remarkable diffraction efficiency performances. In spite of low theoretical DEs and low signal-to-noise ratios owing to the absence of a beamstop, it was possible to perform scanning X-ray microscopy even at 1.5 keV thanks to the precision of the fabrication methods through which the ML-FZPs were fabricated. Furthermore, it was shown by this experiment that a FZP with *A*_*r*_ = 169 can perform very well with hard and soft X-rays, and can resolve at least 60 nm-sized features. As a future work the ultimate resolution of the present ML-FZPs with hard X-rays needs to be determined through rigorous testing in an optimized set-up for higher signal-to-noise ratio. For future work and development of ML-FZPs an important point to be considered is the appropriate choice of material. At this point it has to be noted that the selection of the pair Al_2_O_3_/Ta_2_O_5_ in this work was not made for optimized DE at a specific wavelength but was a compromise between availability and ease of deposition *via* ALD and reasonable DE. Future possibilities for materials choices and resulting achievable resolutions at reasonably high efficiencies will be discussed in the next section in the light of theoretical calculations.

## 4. Materials selection and performance expectations for future ML-FZPs

In addition to the rather reasonable DE of Al_2_O_3_/Ta_2_O_5_ both with soft (Mayer *et al.*, 2011 ▶) and hard X-rays, these materials were essentially selected for their established deposition routes *via* ALD. Nevertheless, the choice of material can be optimized. In this section the optimization of material selection for the hard X-ray range from 8 to 17 keV based on theoretical considerations and current advances in ALD deposition and the availability of materials will be discussed.

**Figure 6:**
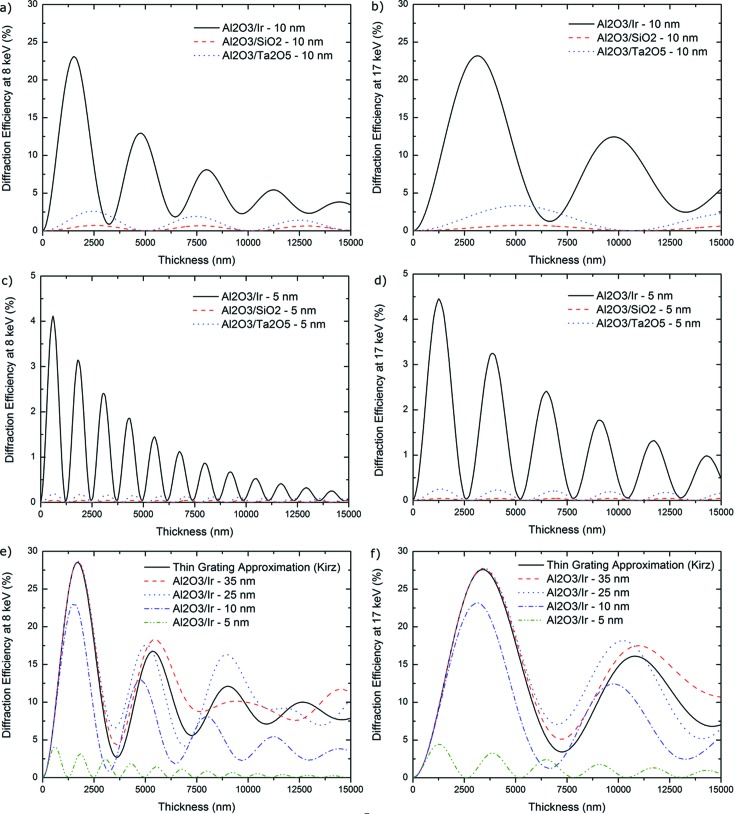
CWT DE calculations of various materials with Δ*r*_*n*_ = 10 nm at (*a*) 8 keV and (*b*) 17 keV and with Δ*r*_*n*_ = 5 nm at (*c*) 8 keV and (*d*) 17 keV. DE calculations according to CWT and the thin-grating approximation for Al_2_O_3_/Ir ML-FZPs with various Δ*r*_*n*_, at (*e*) 8 keV and (*f*) 17 keV. CWT calculation parameters: zones parallel to the optical axis, magnification of 10000×, line-to-space ratio of 1:1.

The diffraction behaviour of Fresnel zone plates (and gratings in general) is classified into three regimes (Maser & Schmahl, 1992 ▶; Moharam & Gaylord, 1981 ▶; Klein & Cook, 1967 ▶); (i) the Raman–Nath (Raman & Nath, 1935 ▶) regime, where the zone plate behaves like a thin grating and where, for instance, the theory developed by Kirz is applicable (Kirz, 1974 ▶); (ii) the Bragg regime, in which the FZP behaves like a thick grating; and (iii) the intermediate diffraction regime. The FZPs that fall into the Raman–Nath regime are characterized by very large Δ*r*_*n*_ relative to wavelength and very low aspect ratio. On the other hand, FZPs with Δ*r*_*n*_ comparable with the wavelength (or even smaller) and high thicknesses fall into the Bragg regime. The third group of FZPs cluster in the intermediate diffraction regime. Most of the high-resolution X-ray FZPs as well as the ML-FZPs presented in this work belong to this latter category (Maser & Schmahl, 1992 ▶). In this case and in the Bragg regime, volume effects within the FZP have to be taken into consideration to account for the diffraction behaviour whereupon it was also shown (Maser & Schmahl, 1992 ▶; Schneider, 1997 ▶) that high resolutions (*e.g.* 10 nm) at high efficiencies can essentially only be achieved if the zones are tilted towards the optical axis to locally satisfy the Bragg condition. Here, we show that an appropriate choice of materials allows this limitation to be circumvented to some extent. Depending on the materials, a reasonable DE can be obtained at hard X-rays without tilting zones for resolutions as low as 10 nm and possibly 5 nm. As an illustration, the DEs of three different material pairs were evaluated within the framework of CWT, which takes the above-mentioned volume effects into account, and are compared in Figs. 6(*a*) and 6(*b*) ▶ for Δ*r*_*n*_ = 10 and in Figs. 6(*c*) and 6(*d*) ▶ for Δ*r*_*n*_ = 5 nm at 8 and 17 keV, respectively. The DEs of the specific pair Al_2_O_3_/Ir at 8 and 17 keV for Δ*r*_*n*_ = 35 to 5 nm are also compared in Figs. 6(*e*) and 6(*f*) ▶.

In a previous study (Mayer *et al.*, 2011 ▶) the pair Al_2_O_3_/SiO_2_ was shown to be a very promising candidate for efficient focusing of soft X-rays. At harder X-rays it appears that Al_2_O_3_/Ir performs very well and has a significantly higher theoretical DE both at 8 and 17 keV photon energies with more than 20% DE for Δ*r*_*n*_ = 10 nm [Figs. 6(*a*) and 6(*b*) ▶]. At even smaller outermost zone widths the CWT predicts reasonable efficiencies with, for instance, >4% for Δ*r*_*n*_ = 5 nm [Fig. 6(*c*) and 6(*d*) ▶]. However, it has to be noted that for such thin zones the resolution of the zone plate may differ from and be effectively lower than that predicted by the Rayleigh criterion (Kang *et al.*, 2006 ▶; Schroer, 2006 ▶). The theoretical DEs of the promising Al_2_O_3_/Ir pair for various Δ*r*_*n*_ are given in Fig. 6(*e*) ▶ for 8 keV and Fig. 6(*f*) for 17 keV. The degradation of the DE when the outermost zone width is decreased from 35 to 10 nm in an Al_2_O_3_/Ir ML-FZP at both energies is not as significant as it is in the case of Al_2_O_3_/Ta_2_O_5_ (Fig. 4 ▶) and renders this material pair very interesting. The preparation of a FZP with very thin zones such as 10 nm and/or presenting a tilt angle towards the optical axis which, as previously mentioned, may be necessary to achieve even higher resolution, are paramount challenges as current fabrication techniques can barely achieve structure sizes below 10 nm. It is believed by the authors that the ALD/FIB technique has the potential to push these boundaries in the future. From a fabrication point of view, both alumina (Niinistö *et al.*, 2004 ▶) and iridium (Aaltonen *et al.*, 2004 ▶) can be deposited *via* established ALD routes. It is also worth noting that *t*_Optimum_ of all above-mentioned Al_2_O_3_/Ir ML-FZPs are of the order of only a few micrometers, which would help in reducing the impact of the previously mentioned effect of structural error accumulation in propagation direction as thickness increases.

## 5. Conclusions

Efficient focusing of 8 keV hard X-rays using ML-FZPs was successfully demonstrated using ML-FZPs with Δ*r*_*n*_ = 10 and 35 nm. Efficiencies up to >15%, representing 77% of theoretically predicted values, were achieved. This was made possible by utilizing high-precision fabrication methods with control over the nanoscale multilayer thickness *via* atomic layer deposition and the microscale structure thickness *via* focused ion beam milling. Soft X-ray scanning microscopy with the ZP35t multilayer Fresnel zone plate showed that the imaging was possible, despite the very low theoretical efficiency and the lack of a beamstop which contributed to the noise in the image, by resolving features as small as 60 nm. Further developments towards efficient and high-resolution diffractive optics considering different materials were discussed using coupled wave theory (CWT) and the thin-grating (TG) approximation. It was shown that use of absorptive iridium and transparent alumina in a multilayer Fresnel zone plate could lead to a theoretical efficiency of more than 20% for an outermost zone width of 10 nm at 8 and 17 keV photon energies. As future ML-FZP materials, Al_2_O_3_/Ir promises reasonably high efficiencies even without introducing a tilt angle to the zones to satisfy the Bragg condition at least down to about 10 nm resolution in the hard X-ray range. Further experiments to test these theoretical predictions are planned for the future.

## Figures and Tables

**Table 1:** Summary of properties of the ML-FZPs ZP35t: ML-FZP with 35 nm outermost zone width and less than optimum thickness. ZP35opt: ML-FZP with 35 nm outermost zone width and thickness close to optimum thickness. ZP10: ML-FZP with 10 nm outermost zone width. *r*_T_/*r*_Act_: total radius of the FZP/width of the active area. 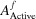: area fraction of active zones. Δ*r*_*n*_: outermost zone width. *N*: number of zones in the active area. *f*_8keV_: focal distance at 8 keV. *t*_Optimum_/*t*_Real_: optimum thickness according to CWT/realised thickness. *A*_*r*_: realised aspect ratio, *t*_Real_/Δ*r*_*n*_.

Type	*r*_T_/*r*_Act_ (µm)	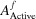 (%)	Δ*r*_*n*_ (nm)	*N*	*f*_8keV_ (µm)	*t*_Optimum_/*t*_Real_ (µm)	*A*_*r*_
ZP35t	19/4	37.7	35	103	∼8581	8.85/∼5.9	∼169
ZP35opt	19/4	37.7	35	103	∼8581	8.85/∼8.5	∼243
ZP10	19/4	37.7	10	360	∼2452	2.45/∼1.9	∼190
